# A blood RNA transcriptome signature for COVID-19

**DOI:** 10.1186/s12920-021-01006-w

**Published:** 2021-06-11

**Authors:** Philip Kam Weng Kwan, Gail B. Cross, Claire M. Naftalin, Bintou A. Ahidjo, Chee Keng Mok, Felic Fanusi, Intan Permata Sari, Siok Ching Chia, Shoban Krishna Kumar, Rawan Alagha, Sai Meng Tham, Sophia Archuleta, October M. Sessions, Martin L. Hibberd, Nicholas I. Paton

**Affiliations:** 1 Department of Medicine, Yong Loo Lin School of Medicine, National University Health System, National University of Singapore, Singapore, Singapore; 2 Division of Infectious Diseases, Department of Medicine, National University Hospital, National University Health System, Singapore, Singapore; 3 Department of Microbiology and Immunology, Yong Loo Lin School of Medicine, National University Health System, National University of Singapore, Singapore, Singapore; 4 Biosafety Level 3 Core Facility, Yong Loo Lin School of Medicine, National University Health System, National University of Singapore, Singapore, Singapore; 5 Department of Pharmacy, National University of Singapore, Singapore, Singapore; 6 London School of Hygiene and Tropical Medicine, London, UK; 7 Infectious Diseases Translational Research Programme, National University of Singapore, Singapore, Singapore; 8 Infectious Diseases Translational Research Programme and Department of Medicine, Yong Loo Lin School of Medicine, National University of Singapore, NUHS Tower Block Level 10, 1E Kent Ridge Road, Singapore, 119228 Singapore

**Keywords:** SARS-CoV-2, COVID-19, Gene expression, Biomarkers, RNA sequencing, Whole blood

## Abstract

**Background:**

COVID-19 is a respiratory viral infection with unique features including a more chronic course and systemic disease manifestations including multiple organ involvement; and there are differences in disease severity between ethnic groups. The immunological basis for disease has not been fully characterised. Analysis of whole-blood RNA expression may provide valuable information on disease pathogenesis.

**Methods:**

We studied 45 patients with confirmed COVID-19 infection within 10 days from onset of illness and a control group of 19 asymptomatic healthy volunteers with no known exposure to COVID-19 in the previous 14 days. Relevant demographic and clinical information was collected and a blood sample was drawn from all participants for whole-blood RNA sequencing. We evaluated differentially-expressed genes in COVID-19 patients (log2 fold change ≥ 1 versus healthy controls; false-discovery rate < 0.05) and associated protein pathways and compared these to published whole-blood signatures for respiratory syncytial virus (RSV) and influenza. We developed a disease score reflecting the overall magnitude of expression of internally-validated genes and assessed the relationship between the disease score and clinical disease parameters.

**Results:**

We found 135 differentially-expressed genes in the patients with COVID-19 (median age 35 years; 82% male; 36% Chinese, 53% South Asian ethnicity). Of the 117 induced genes, 14 were found in datasets from RSV and 40 from influenza; 95 genes were unique to COVID-19. Protein pathways were mostly generic responses to viral infections, including apoptosis by P53-associated pathway, but also included some unique pathways such as viral carcinogenesis. There were no major qualitative differences in pathways between ethnic groups. The composite gene-expression score was correlated with the time from onset of symptoms and nasal swab qPCR CT values (both *p* < 0.01) but was not related to participant age, gender, ethnicity or the presence or absence of chest X-ray abnormalities (all *p* > 0.05).

**Conclusions:**

The whole-blood transcriptome of COVID-19 has overall similarity with other respiratory infections but there are some unique pathways that merit further exploration to determine clinical relevance. The approach to a disease score may be of value, but needs further validation in a population with a greater range of disease severity.

**Supplementary Information:**

The online version contains supplementary material available at 10.1186/s12920-021-01006-w.

## Background

Coronavirus disease 2019 (COVID-19), caused by the severe acute respiratory syndrome coronavirus 2 (SARS-CoV-2), was first identified in China in December 2019 and has since progressed to cause a major global pandemic [1, 2]. Although COVID-19 shares some clinical manifestations with other respiratory viral infections such the common cold (rhinoviruses and common human coronaviruses) and influenza, it has a number of differences including prolonged viral shedding that may last many weeks, progression to more severe disease in a proportion of patients in the second week of illness, and extrapulmonary manifestations including cardiovascular and thromboembolic disease [3, 4]. The disease differs in frequency and severity between ethnic groups and although these differences are most likely driven by socioeconomic factors, it has been suggested that biological factors may also contribute [5, 6].

Key features of the immune response to the virus are lymphopenia (possibly caused by apoptosis via the P53-signaling pathway in T-lymphocytes [7], via angiotensin-converting enzyme (ACE2) protein receptor) [8], and an increase in inflammatory cytokines (cytokine storm) such as interferon-gamma (IFN-γ) and interleukin (IL)-6 [9], which can result in multiple-organ dysfunction syndrome (MODS) and acute respiratory distress syndrome (ARDS), a major cause of death in COVID-19 [10].

Transcriptome analysis of whole blood is a useful tool for profiling the host immune response to an infectious disease. The approach has previously been applied to other respiratory infections, and has identified characteristic gene signatures associated with influenza and respiratory syncytial virus (RSV) infection [11, 12]. It has shown particular value in tuberculosis where, in addition to identifying clinical cases [13], it has potential to identify contacts with asymptomatic disease exposure [14], as well as those with latent infection who will progress to develop symptomatic clinical TB disease [15].

We examined the whole blood gene expression profile in patients with COVID-19 disease to assess whether there are differences from other viral respiratory infections; whether there are any differences between ethnicities; and whether the transcriptome varies according to clinical disease manifestations.

## Methods

### Participants and sample collection

The COVID-19 case group were enrolled from the inpatient wards and emergency department of the National University Hospital, Singapore between 1st April and 26th May 2020. Cases were required to be over the age of 18 and meet a virological case definition for COVID-19 (detectable viral gene by PCR in throat and/or nasopharyngeal swab(s); or in a sputum sample taken within the 7 days prior to the study day). Demographic and clinical data were collected including details of exposure; presence of abnormal findings on respiratory examination; whether supplemental oxygen was required; and presence of abnormal findings consistent with infection on a chest X-ray. A whole blood sample was drawn into a Tempus RNA preservation tube (Thermo Fisher Scientific, Massachusetts, USA) and stored at − 80 °C within 2 h from collection until the time of analysis.

The control group was recruited through an established database of healthy volunteers for clinical research studies. They were required to have no history of travel to high-risk areas; no known exposure to a proven or suspected COVID-19 case in the previous 14 days; no upper or lower respiratory tract infection or any other active illness at the time of visit; and no past or current history of serious chronic disease such as autoimmune disease. Whole blood was collected in an RNA preservation tube and stored as described above. Participants were contacted by telephone at 2 and 4 weeks after the study visit to ask about the development of symptoms of COVID-19 after the study visit and any participants with developing symptoms were excluded.

The study was performed in accordance with the Declaration of Helsinki and approved by the Singapore National Healthcare Group Domain Specific Review Board (NHG DSRB; reference code: DSRB 2020/00286), and all participants gave written informed consent.

### Extraction of total RNA from peripheral blood and RNA sequencing

Samples were thawed and total RNA was extracted; DNAse-treatment was performed using the column-based Tempus™ Spin RNA Isolation kit (ThermoFisher Scientific, Massachusetts, USA) in the Biosafety Level 3 (BSL-3) Laboratory at the National University of Singapore (NUS). RNA was quantified using the Agilent 2100 Bioanalyzer (Agilent Technologies, California, USA). Complementary DNA (cDNA) libraries were constructed using the NEBNext ® poly-(A) mRNA Magnetic Isolation Module and Ultra ™ Directional RNA Library Prep kit (New England Biolabs, Massachusetts, USA). RNA sequencing was performed on Illumina Novaseq 6000 (2 × 151 bp) at NovogeneAIT Genomics Singapore Pte Ltd, Singapore.

### RNASeq data analyses and functional annotation

Sequenced reads (paired-end FASTQ files) were mapped to the Genome Reference Consortium Human Build 38 release 86 (GRCh38.r86) by using STAR aligner (version 2.3.0e) [16]. The aligned reads were counted for each gene using HTSeq (version 0.6.1) [17]. Sample read counts were adjusted for library size and normalized using Trimmed Mean of M-values (TMM) method and multidimensional scaling plot was used to detect any outlier samples (none found; Additional file 1: Fig. 1) using Bioconductor package EdgeR (version 3.18.1) [18].

Gene expression in the COVID-19 case group was compared with the healthy control group using EdgeR (3.18.1). All bioinformatics parameters were according to the standard instructions for gene expression analysis (Additional file 1: Fig. 2). Genes were considered differentially expressed if they had a false-discovery rate (FDR, Benjamini-Hochberg) [19] of less than 0.05 and at least a log2 fold change of ± 1. The nature of the differentially expressed genes was explored further by creating a comprehensive network representation of the proteins associated with the genes in the signature using Search Tool for the Retrieval of Interacting Genes/Proteins (STRING; version 11.0b) [20]. Analysis of the associated functional pathways was performed using the GeneOntology (go) and Kyoto Encyclopedia of Genes and Genomes (KEGG) database on STRING using default settings (false-discovery rate < 0.05).

The list of differentially expressed genes was compared with lists of genes reported in previous whole-blood gene expression studies in patients with Influenza or respiratory syncytial virus (RSV) infection (FDR < 0.05, log2 fold change of ± 1) [11, 12], and the overlap was tested using hypergeometric probability. The pathway analysis was performed in the same way for RSV and influenza from the published gene lists [11, 12].

The analysis of differential gene expression was repeated with stratification by Chinese or South Asian ethnicity (i.e. differential expression of genes in cases compared to controls, both groups limited to a single ethnicity) and analysis of pathways. The association between the relative magnitudes of overexpression of individual genes in the two ethnic groups was assessed by Spearman rank correlation and the difference by Wilcoxon signed-rank test.

To evaluate the relationship between a gene expression profile and demographic and clinical factors we first reduced the list of genes to a smaller, more robust core by splitting cases and controls randomly into two datasets using the python software’s “random ()” function. The differential analysis comparing cases versus controls, as described above, was repeated separately in the two datasets and the induced genes that overlapped between both sets were taken as the “final disease signature”. A disease score was calculated for each COVID-19 case using the normalized gene expression values of the genes in the final disease signature, following an approach described previously [21–23]. Briefly, the disease scores for each patient sample were computed by taking the difference between normalized gene expression values of all induced genes and repressed genes (both relative to controls).

The purpose of the disease score method is to assess the relationship between the final disease signature and clinical parameters. The relationship between this disease score and various dichotomous variables including sex; age (above or below median); ethnicity (South Asian versus Chinese); presence or absence of chest X-ray (CXR) abnormality; time from onset of illness (above or below the median); and quantitative PCR cycle threshold (qPCR CT) from the nasopharyngeal/throat (hereafter termed “nasal”) swab taken closest to time of the study blood draw (limited to 1 week before or after the blood draw; CT value above or below median) was assessed by Mann–Whitney U test. The relationship between the disease score and time from disease onset or qPCR CT, each expressed as continuous variables, was evaluated using Spearman’s rank correlation. All analyses and figures were generated using the R software or custom Python scripts.

The sample size for COVID-19 cases and healthy controls was determined by pragmatic considerations of the feasibility of sample collection given clinical constraints at the time of conducting the study and by previous experience of other host transcriptome studies in infectious diseases where groups of approximately 20–50 individuals typically allow detection of robust differential gene expression in a study group of interest.

All methods were performed in accordance with the relevant guidelines and regulations.

## Results

All extracted RNA samples passed the quality control requirements (RNA integrity number > 7) for RNA sequencing, and a minimum of 85 million raw sequencing reads (Additional file 1: Table 1). On average, 98% of the reads were successfully mapped to the human genome GRCh38.r86 (Additional file 1: Table 2).

We enrolled 45 COVID-19 cases (82% male; median 35 years; 36% Chinese, 53% South Asian, 11% other ethnicity) and 19 healthy controls (58% male; mean age 31 years; 53% Chinese, 47% South Asian). The median duration of illness (based on symptoms in 43 patients) was 4 days (range 1–10 days) prior to the study day. The median qPCR CT value of nasal swabs (CT values available in 35 patients) was 25 (range 13–37). Fourteen COVID-19 (31%) cases had abnormal chest X-rays, 5 (11%) had abnormal respiratory examination findings and 4 (8%) required respiratory support (2 required supplemental oxygen and 2 required mechanical ventilation) at the time of the study.

We found 135 differentially-expressed protein-coding genes (117 induced, 18 repressed) in COVID-19 cases compared to healthy controls (Additional file 1: Table 3). Of these, 16 (14 induced, 2 repressed; Additional file 1: Fig. 3) have been reported previously in RSV infection; 40 (all induced; Additional file 1: Fig. 4) have been reported previously in influenza infection (fold change values of overlap genes were not associated between COVID-19 and either or the two infections); and 13 (induced) in both infections (Additional file 1: Tables 4–5; *p* < 0.01 for overlap) [11, 12]. Analysis of the COVID-19 induced genes by GO identified 141 pathways of which 111, mostly representing generic viral infection and inflammation pathways, were found in one or both of RSV [11] and Influenza [12] (Additional file 1: Table 4). However, 30 pathways were unique to COVID-19 patients, including protein pathways related to P53 apoptosis (Additional file 1: Table 6). Analysis of induced genes using KEGG revealed 11 pathways, 7 of which were found in one or both of RSV [11] and influenza [12] (Additional file 1: Table 7). Four KEGG pathways were unique to COVID-19, including the pathways of viral carcinogenesis and acute myeloid leukemia. No enriched GO or KEGG pathways were found for the 31 repressed genes.

We identified 148 genes (122 induced, 26 repressed) that were differentially expressed in cases of South Asian ethnicity (n = 24; 23 male, 1 female cases versus 5 male, 4 female controls) and 297 genes (170 induced, 127 repressed) that were differentially expressed in cases of Chinese ethnicity (n = 16; 11 male, 5 female cases versus 6 male, 4 female controls) compared to their respective ethnicity-matched controls. There were 76 genes (69 induced, 7 repressed) common to both ethnicities; 221 genes that were unique to Chinese and 72 genes that were unique to South Asian ethnicity. Pathway analysis of these unique genes revealed 78 enriched GO and 3 KEGG pathways in Chinese (Additional file 1: Table 8) and 21 enriched GO pathways and no enriched KEGG pathways in South Asian patients (Additional file 1: Table 9), mostly related to immune and inflammatory responses with no major qualitative difference in types of responses between the two ethnic groups.

**Fig. 1:**
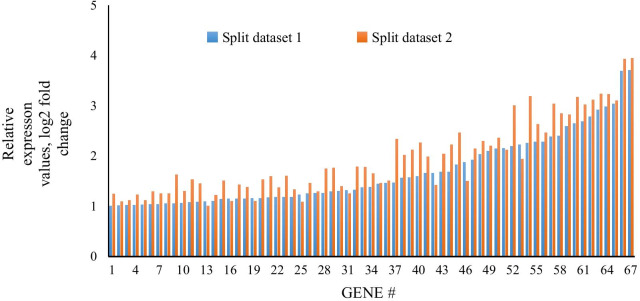
Relative expression of the 67 induced genes in both randomly split datasets. Overall pattern similar between independent comparisons (Spearman rank *p* < 0.0001; r = 0.92)

The final COVID-19 disease signature, obtained by reduction in two randomly split datasets, comprised 67 induced protein-coding genes (all present in the analysis of cases as a single group; Fig. 1), associated with 82 GO and 10 KEGG pathways. These pathways included 75 GO and 8 KEGG pathways enriched in the single group analysis; and 10 GO and 2 KEGG pathways (including viral carcinogenesis and acute myeloid leukemia) that were identified as unique to COVID-19 in the single-group analysis (Additional file 1: Table 10).

**Fig. 2:**
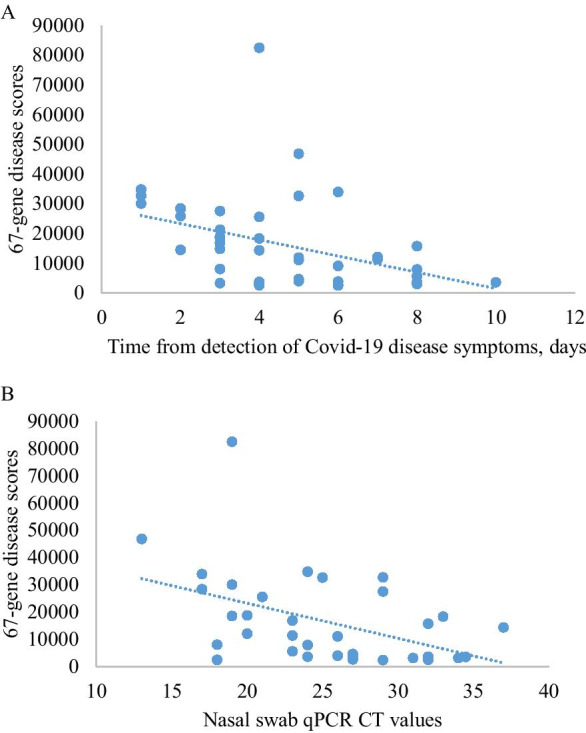
**A** relationship between scores and onset (days) of COVID infection for each patient. Spearman rank correlation, rho =  − 0.49, *p* = 0.001. **B** Relationship between scores and nasal swab qPCR CT values. Spearman rank correlation test, rho =  − 0.48; *p* = 0.004

The median COVID-19 disease score, based on the magnitude of expression of these 67 genes in the final disease signature, was significantly higher in cases studied less than 4 days after illness onset (Table 1, Fig. 2A); and in cases with nasal swab qPCR CT values below 25 (Table 1, Fig. 2B). The score did not differ by other factors examined (Table 1).

**Table 1:** Summary of association tests on disease scores for clinical parameters in Covid-19 cases. Scores are tested for significance using Mann–Whitney U test,

Clinical parameters in Covid-19 cases	N (%)*	Median score [range]	*p* value
*Age*			0.67
< 35 years	22(51)	14,415 [3047–46,834]	
≥ 35 years	23(49)	11,116 [2394–82,442]	
*Gender*			0.11
Male	37 (82)	11,116 [2394–46,834]	
Female	8 (18)	26,542 [3180–82,442]	
Ethnicity			0.18
Chinese	16 (36)	4991 [2393–82,442]	
South Asian	24 (53)	15,283 [2522–46,8234]	
*Chest X-ray*			0.69
Abnormal	14 (31)	10,109 [2596–82,442]	
Normal	29 (54)	14,474 [2394–46,834]	
*Days from onset of COVID infection*			0.003
< 4 days	15 (33)	21,265 [3327–34,810]	
≥ 4 days	28 (62)	6759 [2522–82,442]	
*Nasal swab qPCR CT values*			0.014
< 25	17 (38)	18,547 [2522–82,442]	
≥ 25	18 (40)	3928 [2394–32,696]	

*% is using denominator of those with available data

## Discussion

We identified a whole-blood RNA expression signature for COVID-19 patients comprising 135 differentially-expressed, protein-coding genes. This signature is biologically plausible as a signature for COVID-19 disease as many of the genes overlap those previously published for other respiratory viral diseases, in particular influenza and RSV, and contains many enriched pathways for generic responses to respiratory viral infections such as Type-1 interferon and inflammatory responses [24]. The finding of enriched pathways for P53 is consistent with a previous small study of RNA expression in peripheral blood mononuclear cell (PMBC) from patients with COVID-19 [7]. Although we did not find evidence for enrichment of this pathway in our analysis using published whole blood transcriptome gene lists for influenza and RSV, P53 is known to be important in many viral infections [25], inducing apoptosis in virus-infected cells to limit viral replication [26, 27]. We also found enrichment of several cancer pathways (viral carcinogenesis and acute myeloid leukemia; *BCL2A1, FCGR1A, JUP, PML* and *CDKN1A, HIST1H2BD, HIST1H2BJ, IRF7* respectively), which have not been described in previous acute respiratory virus infections in the published literature. This raises the possibility that COVID-19 may have oncogenic effects, a phenomenon well established in chronic viral infections such as EBV infection which has potent lymphocyte growth transforming activity that can predispose to a variety of hematological malignancies [28]. This finding should be treated with caution—it needs to be validated in independent studies and expression of these pathways may not translate to an increase in cancer incidence. However, COVID-19 follows a more chronic course and with more widespread systemic manifestations than other acute respiratory viral infections [3]; and there are longer-term consequences of this infection that are only just beginning to be described [29]. Any predisposition to malignancy could take many years to become apparent; it is a vital area for future research given the scale of the COVID-19 pandemic. We sought but did not find evidence of enrichment of organ-specific pathways that may be relevant to COVID-19 pathogenesis. In particular we did not find any related to ACE-2 receptor genes (common in lung and heart epithelium tissues) which is known to be the route of entry of SARS-Cov-2 into cells [8], although it is not surprising this is absent in the whole-blood transcriptome given that ACE2 is not commonly expressed in immune cells; gene expression studies (based on study of specific cellular receptor genes) in other tissues might yield different results [30].

A number of studies have described inter-ethnic differences in the risk of acquiring COVID-19 and the risk of progression to more severe disease, findings which are likely to be explained by socioeconomic factors [6]. We found a number of differences in the profile of gene expression and the associated protein pathways between the two major ethnic groups in our dataset, but these were mostly in generic pathways expected in the immune response to infection which likely represent variation associated with the relatively small sub-group sample sizes rather than meaningful inter-ethnic differences. The two ethic groups studied are both at the lower end of the range of COVID-19 disease severity compared with other ethnic groups, based on epidemiological (non-transcriptomic) data in the UK population [6, 31, 32], and comparison of outcomes between similar ethnic groups in Malaysia did not show a difference [33].

There is a strong clinical need for a test that could indicate underlying disease severity and predict those with mild COVID-19 disease who are most at risk of progressing to severe forms of disease. Given that the progression is most likely driven by the immune response rather than the virus per se, an immune-based test may hold the best promise for this indication. As a first step, we evaluated whether a quantitative disease score based on the magnitude of gene expression was related to clinical parameters. Our finding that the score was associated with the time from illness onset (score lower with greater time from onset of illness) and CT (qPCR) on nasal swabs (score higher with greater viral burden on the nasal swab) suggest initial biological plausibility for the responsiveness of the score to clinical disease/viral burden. Other studies have shown an independent relationship between qPCR CT and disease outcome [34–38]. We did not find any direct relationship between the disease score and indicators of disease severity. However, we studied a group of patients with relatively mild disease and the chest X-ray we used to assess disease severity is an imprecise measure that may underestimate the extent of lung pathology. Given the heterogeneity of clinical presentation a much larger study would be required to address this including greater representation of patients with more severe disease and with better markers of disease severity such a pulmonary CT imaging. This evaluation was intended as an initial proof of concept that an integrated measure of gene expression (the risk score) might relate to clinical disease parameters—for this score to be clinically useful it would need to be reduced to a smaller number of genes that could be measured using a simple test (such as lateral flow) and would need to be shown to improve the accuracy of prediction of those at risk of disease progression over and above the prediction available from readily-available clinical parameters.

The main limitation of our study is the relatively small sample size, although it proved sufficient to identify a signature and analyse associated pathways. However, the gene signature requires further validation in an independent dataset. The number of genes in our final signature is relatively large and for the approach to have potential clinical use as a rapid test, further reduction to a small number of key genes by RT-PCR would be required. The healthy controls were selected on the basis of absence of exposure to COVID-19 and absence of symptoms; we did not perform a nasal swab PCR to detect asymptomatic carriage of COVID-19. However, at the time of the study community transmission of COVID-19 in Singapore was at a very low rate (approximately 35 new cases per day, in a local community population of 4 million) at the time the study was conducted [39], and we followed the participants for four weeks after the study to rule out subsequent development of symptomatic disease. Even if a small proportion of the controls had undetected infection, this would not explain the findings of differential gene expression in the COVID-19 cases—if anything the bias would be towards decreasing the level of relative gene expression. Clinical data collection in COVID-19 cases was necessarily limited due to constraints of patient access and investigation accompanying stringent institutional infection control protocols. Similarly, constraints on laboratory access and sample transportation meant that we could not collect and process other blood samples for a more comprehensive immunological profile that might provide additional context for the transcriptome findings.

## Conclusions

In conclusion, we have measured a whole-blood transcriptome in patients with COVID-19 that has indicated the possible value of this approach for further characterizing disease pathogenesis and the host response to infection.

## Supplementary Information

**Additional file 1.** Supplementary tables and figures.

## Data Availability

The datasets generated and/or analysed during the current study are available in NCBI Sequencing Read Archive under the BioProject accession PRJNA692253 (http://www.ncbi.nlm.nih.gov/bioproject/692253).

## Abbreviations

CT: Cycle threshold
CXR: Chest X-ray
GO: Gene Ontology
KEGG: Kyoto Encyclopedia of Genes and Genomes
PBMC: Peripheral blood mononuclear cell
RSV: Respiratory syncytial virus
