# Supplementary material for: A blood RNA transcriptome signature for COVID-19
Source: BMC Med Genomics. 2021 Jun 11;14:155. doi: 10.1186/s12920-021-01006-w (PMC8193593; doi:10.1186/s12920-021-01006-w)
Supplement: Supplementary file 1 — Additional file 1. Supplementary tables and figures. [file 12920_2021_1006_MOESM1_ESM.docx]

Supplementary materials

Manuscript title: A blood RNA transcriptome signature for COVID-19

Philip Kam Weng Kwan^a^

Gail B. Cross^a,b^

Claire M. Naftalin^a^

Bintou A. Ahidjo^c,d^

Chee Keng Mok^c,d^

Felic Fanusi^c^

Intan Permata Sari^a^

Siok Ching Chia^a^

Shoban Krishna Kumar^b^

Rawan Alagha^b^

Sai Meng Tham^b^

Sophia Archuleta^a,b^

October M. Sessions^e^

Martin L. Hibberd^a,f^

Nicholas I. Paton^a,b,f#^

^a^Department of Medicine, Yong Loo Lin School of Medicine, National University Health System, National University of Singapore

^b^Division of Infectious Diseases, Department of Medicine, National University Hospital, National University Health System, Singapore

^c^Department of Microbiology and Immunology, Yong Loo Lin School of Medicine, National University Health System, National University of Singapore

^d^Biosafety Level 3 Core Facility, Yong Loo Lin School of Medicine, National University Health System, National University of Singapore

^e^Department of Pharmacy, National University of Singapore

^f^London School of Hygiene & Tropical Medicine, United Kingdom

**SUPPLEMENTARY TABLES AND FIGURES**

Supplementary Table 1. Quality control results for all 64 RNA sequencing samples.

| Sample | Raw reads | Raw data(G) | Effective(%) | Error(%) | Q20(%) | Q30(%) | GC(%) |
| --- | --- | --- | --- | --- | --- | --- | --- |
| S_11X | 95575204 | 14.3 | 98 | 0 | 98 | 95 | 56 |
| S_12X | 100186506 | 15 | 99 | 0 | 98 | 95 | 55 |
| S_14X | 111108536 | 16.7 | 98 | 0 | 98 | 95 | 57 |
| S_15X | 129189732 | 19.4 | 98 | 0 | 98 | 94 | 54 |
| S_17X | 99028422 | 14.9 | 98 | 0 | 98 | 96 | 57 |
| S_18X | 96759952 | 14.5 | 98 | 0 | 98 | 95 | 56 |
| S_19X | 110119346 | 16.5 | 98 | 0 | 98 | 95 | 55 |
| S_1X | 116864648 | 17.5 | 99 | 0 | 98 | 95 | 56 |
| S_20X | 111073532 | 16.7 | 99 | 0 | 98 | 94 | 53 |
| S_21X | 89138430 | 13.4 | 98 | 0 | 98 | 95 | 53 |
| S_22X | 95890404 | 14.4 | 98 | 0 | 98 | 95 | 56 |
| S_24X | 93362238 | 14 | 98 | 0 | 98 | 96 | 57 |
| S_27X | 109737830 | 16.5 | 99 | 0 | 98 | 95 | 57 |
| S_28X | 106602634 | 16 | 98 | 0 | 98 | 95 | 54 |
| S_29X | 106899630 | 16 | 98 | 0 | 98 | 95 | 56 |
| S_2X | 126901398 | 19 | 99 | 0 | 98 | 95 | 57 |
| S_31X | 106019810 | 15.9 | 98 | 0 | 98 | 95 | 57 |
| S_32X | 130897024 | 19.6 | 97 | 0 | 98 | 96 | 57 |
| S_33X | 102981088 | 15.4 | 98 | 0 | 98 | 95 | 55 |
| S_35X | 107857464 | 16.2 | 99 | 0 | 98 | 95 | 54 |
| S_36X | 115917998 | 17.4 | 98 | 0 | 98 | 95 | 57 |
| S_37X | 109938210 | 16.5 | 99 | 0 | 98 | 95 | 56 |
| S_38X | 99213350 | 14.9 | 98 | 0 | 98 | 95 | 58 |
| S_39X | 112980310 | 16.9 | 98 | 0 | 98 | 95 | 54 |
| S_3X | 110467382 | 16.6 | 99 | 0 | 98 | 95 | 55 |
| S_40X | 137266670 | 20.6 | 99 | 0 | 98 | 94 | 53 |
| S_41X | 101517142 | 15.2 | 98 | 0 | 98 | 95 | 55 |
| S_42X | 101838514 | 15.3 | 99 | 0 | 98 | 95 | 54 |
| S_44X | 125908076 | 18.9 | 98 | 0 | 98 | 95 | 55 |
| S_45X | 95131000 | 14.3 | 98 | 0 | 98 | 95 | 55 |
| S_46X | 86557024 | 13 | 99 | 0 | 98 | 95 | 54 |
| S_47X | 132286546 | 19.8 | 99 | 0 | 98 | 95 | 54 |
| S_49X | 101337968 | 15.2 | 97 | 0 | 98 | 95 | 56 |
| S_4X | 100360906 | 15.1 | 99 | 0 | 99 | 96 | 57 |
| S_50X | 106478972 | 16 | 97 | 0 | 98 | 95 | 56 |
| S_51X | 110178548 | 16.5 | 97 | 0 | 98 | 95 | 56 |
| S_53X | 105297622 | 15.8 | 97 | 0 | 98 | 95 | 56 |
| S_54X | 135535052 | 20.3 | 97 | 0 | 98 | 96 | 58 |
| S_55X | 136948878 | 20.5 | 97 | 0 | 99 | 96 | 58 |
| S_58X | 159014854 | 23.9 | 98 | 0 | 98 | 95 | 54 |
| S_60X | 117231332 | 17.6 | 98 | 0 | 98 | 95 | 54 |
| S_62X | 139106196 | 20.9 | 98 | 0 | 98 | 95 | 57 |
| S_64X | 161763640 | 24.3 | 98 | 0 | 98 | 95 | 56 |
| S_66X | 150873942 | 22.6 | 98 | 0 | 98 | 95 | 55 |
| S_68X | 130295336 | 19.5 | 98 | 0 | 98 | 95 | 57 |
| S_69X | 136373576 | 20.5 | 98 | 0 | 98 | 95 | 56 |
| S_6X | 123564038 | 18.5 | 99 | 0 | 98 | 95 | 55 |
| S_70X | 140233344 | 21 | 98 | 0 | 98 | 95 | 55 |
| S_71X | 152745988 | 22.9 | 98 | 0 | 98 | 95 | 55 |
| S_72X | 135234218 | 20.3 | 98 | 0 | 98 | 95 | 56 |
| S_73X | 133503934 | 20 | 98 | 0 | 98 | 95 | 59 |
| S_75X | 113490294 | 17 | 99 | 0 | 98 | 95 | 54 |
| S_76X | 166421822 | 25 | 98 | 0 | 99 | 96 | 56 |
| S_77X | 121879436 | 18.3 | 98 | 0 | 99 | 96 | 59 |
| S_78X | 147733184 | 22.2 | 98 | 0 | 98 | 95 | 58 |
| S_7X | 136242394 | 20.4 | 99 | 0 | 98 | 95 | 56 |
| S_80X | 149136838 | 22.4 | 96 | 0 | 98 | 95 | 58 |
| S_84X | 165424530 | 24.8 | 98 | 0 | 99 | 96 | 58 |
| S_85X | 115117958 | 17.3 | 98 | 0 | 98 | 95 | 53 |
| S_86X | 146414324 | 22 | 99 | 0 | 98 | 95 | 57 |
| S_87X | 109366692 | 16.4 | 99 | 0 | 98 | 95 | 58 |
| S_89X | 127689326 | 19.2 | 96 | 0 | 98 | 95 | 55 |
| S_8X | 114659772 | 17.2 | 99 | 0 | 98 | 95 | 54 |
| S_91X | 134166306 | 20.1 | 98 | 0 | 98 | 95 | 53 |

Supplementary Table 2. Mapping statistics for all 64 RNA sequencing reads via STAR bioinformatics software.

| ID | Input reads | Unique reads | %total mapped to reference genome | % unique reads | % mapped to multiple loci | % unmapped: too short |
| --- | --- | --- | --- | --- | --- | --- |
| S_10X | 60713868 | 49758211 | 98 | 82 | 16 | 2 |
| S_11X | 47787602 | 41345932 | 98 | 87 | 11 | 2 |
| S_13X | 49154398 | 44020721 | 98 | 90 | 9 | 2 |
| S_15X | 64594866 | 58698117 | 98 | 91 | 7 | 2 |
| S_17X | 49514211 | 37437587 | 98 | 76 | 23 | 2 |
| S_18X | 48379976 | 41050768 | 98 | 85 | 13 | 2 |
| S_19X | 55059673 | 47511195 | 98 | 86 | 12 | 2 |
| S_1X | 58432324 | 46831342 | 98 | 80 | 18 | 2 |
| S_20X | 55536766 | 45550481 | 85 | 82 | 3 | 15 |
| S_21X | 44569215 | 41590219 | 98 | 93 | 4 | 2 |
| S_22X | 47945202 | 41632735 | 98 | 87 | 11 | 2 |
| S_25X | 48338326 | 42345989 | 98 | 88 | 11 | 1 |
| S_26X | 63095680 | 51839568 | 98 | 82 | 16 | 2 |
| S_27X | 54868915 | 42251755 | 98 | 77 | 21 | 2 |
| S_28X | 53301317 | 47892340 | 98 | 90 | 8 | 2 |
| S_29X | 53449815 | 41299681 | 99 | 77 | 21 | 1 |
| S_2X | 63450699 | 49040312 | 97 | 77 | 20 | 1 |
| S_30X | 67564254 | 54512193 | 98 | 81 | 18 | 2 |
| S_31X | 53009905 | 40665747 | 98 | 77 | 22 | 2 |
| S_37X | 54969105 | 42757050 | 99 | 78 | 21 | 1 |
| S_38X | 49606675 | 33489508 | 98 | 68 | 31 | 2 |
| S_39X | 56490155 | 48274249 | 99 | 85 | 13 | 1 |
| S_3X | 55233691 | 49540698 | 98 | 90 | 9 | 2 |
| S_40X | 68633335 | 59484389 | 98 | 87 | 12 | 2 |
| S_41X | 50758571 | 41423026 | 99 | 82 | 17 | 1 |
| S_42X | 50919257 | 43073945 | 99 | 85 | 14 | 1 |
| S_43X | 65749250 | 58073938 | 98 | 88 | 10 | 2 |
| S_44X | 62954038 | 52543985 | 98 | 83 | 15 | 2 |
| S_45X | 47565500 | 39017605 | 99 | 82 | 17 | 1 |
| S_46X | 43278512 | 37970855 | 98 | 88 | 11 | 2 |
| S_47X | 66143273 | 58197409 | 98 | 88 | 10 | 2 |
| S_52X | 60092476 | 51384990 | 98 | 86 | 13 | 2 |
| S_53X | 52648811 | 42519884 | 98 | 81 | 17 | 2 |
| S_54X | 67767526 | 46216728 | 98 | 68 | 30 | 2 |
| S_55X | 68474439 | 48231915 | 98 | 70 | 28 | 2 |
| S_56X | 70824329 | 59212473 | 98 | 84 | 15 | 2 |
| S_57X | 83459588 | 71380613 | 98 | 86 | 12 | 2 |
| S_58X | 79507427 | 71330700 | 98 | 90 | 9 | 1 |
| S_59X | 70587201 | 62839215 | 98 | 89 | 9 | 2 |
| S_60X | 58615666 | 51948922 | 98 | 89 | 10 | 2 |
| S_61X | 67463207 | 51956029 | 98 | 77 | 21 | 2 |
| S_62X | 69553098 | 51738476 | 98 | 74 | 24 | 2 |
| S_63X | 65133521 | 49651034 | 98 | 76 | 22 | 2 |
| S_64X | 80881820 | 64011636 | 98 | 79 | 19 | 2 |
| S_65X | 67852806 | 58810399 | 98 | 87 | 12 | 2 |
| S_66X | 75436971 | 62629242 | 99 | 83 | 16 | 1 |
| S_67X | 67960034 | 52209467 | 98 | 77 | 22 | 2 |
| S_68X | 65147668 | 51395850 | 98 | 79 | 19 | 2 |
| S_6X | 61782019 | 54572720 | 98 | 88 | 10 | 2 |
| S_74X | 60008849 | 37944195 | 99 | 63 | 35 | 1 |
| S_75X | 56745147 | 51076430 | 98 | 90 | 8 | 2 |
| S_76X | 83210911 | 71379699 | 98 | 86 | 12 | 2 |
| S_77X | 60939718 | 39815873 | 99 | 65 | 33 | 1 |
| S_78X | 73866592 | 54267619 | 98 | 73 | 25 | 2 |
| S_7X | 68121197 | 58424077 | 98 | 86 | 12 | 2 |
| S_80X | 74568419 | 55277492 | 98 | 74 | 24 | 2 |
| S_82X | 87744598 | 62161596 | 98 | 71 | 28 | 1 |
| S_85X | 57558979 | 51119861 | 98 | 89 | 9 | 2 |
| S_86X | 73207162 | 60303973 | 98 | 82 | 16 | 2 |
| S_88X | 79370025 | 60129043 | 99 | 76 | 23 | 1 |
| S_89X | 63844663 | 58566381 | 97 | 92 | 5 | 3 |
| S_8X | 57329886 | 49690343 | 98 | 87 | 12 | 2 |
| S_90X | 72447174 | 53582290 | 98 | 74 | 24 | 2 |
| S_9X | 50972505 | 43101369 | 98 | 85 | 13 | 2 |

Supplementary Table 3. List of the 135 protein-coding-gene COVID-19 signature from the comparison between COVID-19 cases and controls (false discovery rate < 0.05; log2 fold change > 1 or < -1).

| Ensembl_ID | Genename | Gene description | log2FC | logCPM |
| --- | --- | --- | --- | --- |
| ENSG00000091262 | ABCC6 | ATP binding cassette subfamily C member 6 | -1.0 | 6.7 |
| ENSG00000159618 | ADGRG5 | adhesion G protein-coupled receptor G5 | -1.3 | 6.4 |
| ENSG00000148926 | ADM | adrenomedullin | 1.1 | 5.1 |
| ENSG00000188157 | AGRN | agrin | 2.1 | 4.9 |
| ENSG00000140379 | BCL2A1 | BCL2 related protein A1 | 1.4 | 3.7 |
| ENSG00000106605 | BLVRA | biliverdin reductase A | 1.2 | 3.7 |
| ENSG00000130303 | BST2 | bone marrow stromal cell antigen 2 | 1.5 | 6.7 |
| ENSG00000166323 | C11orf65 | chromosome 11 open reading frame 65 | -1.1 | 8.1 |
| ENSG00000122783 | C7orf49 | cell cycle regulator of NHEJ | 1.0 | 10.6 |
| ENSG00000165181 | C9orf84 | shortage in chiasmata 1 | -1.4 | 5.0 |
| ENSG00000141837 | CACNA1A | calcium voltage-gated channel subunit alpha1 A | 1.1 | 4.3 |
| ENSG00000163823 | CCR1 | C-C motif chemokine receptor 1 | 1.3 | 5.3 |
| ENSG00000183625 | CCR3 | C-C motif chemokine receptor 3 | 1.0 | 10.2 |
| ENSG00000134058 | CDK7 | cyclin dependent kinase 7 | -1.4 | 3.6 |
| ENSG00000124762 | CDKN1A | cyclin dependent kinase inhibitor 1A | 1.3 | 4.6 |
| ENSG00000086065 | CHMP5 | charged multivesicular body protein 5 | 1.0 | 3.8 |
| ENSG00000137200 | CMTR1 | cap methyltransferase 1 | 1.1 | 5.3 |
| ENSG00000203667 | COX20 | cytochrome c oxidase assembly factor COX20 | -1.1 | 5.3 |
| ENSG00000104218 | CSPP1 | centrosome and spindle pole associated protein 1 | -1.1 | 3.3 |
| ENSG00000229754 | CXCR2P1 | C-X-C motif chemokine receptor 2 pseudogene 1 | 1.6 | 4.3 |
| ENSG00000073737 | DHRS9 | dehydrogenase/reductase 9 | 1.9 | 3.0 |
| ENSG00000108771 | DHX58 | DExH-box helicase 58 | 1.8 | 5.6 |
| ENSG00000175550 | DRAP1 | DR1 associated protein 1 | 1.0 | 7.4 |
| ENSG00000146425 | DYNLT1 | dynein light chain Tctex-type 1 | 1.1 | 4.4 |
| ENSG00000172889 | EGFL7 | EGF like domain multiple 7 | 1.5 | 2.6 |
| ENSG00000055332 | EIF2AK2 | eukaryotic translation initiation factor 2 alpha kinase 2 | 1.0 | 3.5 |
| ENSG00000132464 | ENAM | enamelin | 1.3 | 9.8 |
| ENSG00000132199 | ENOSF1 | enolase superfamily member 1 | 1.3 | 5.3 |
| ENSG00000133106 | EPSTI1 | epithelial stromal interaction 1 | 2.6 | 5.7 |
| ENSG00000149564 | ESAM | endothelial cell adhesion molecule | 1.0 | 3.6 |
| ENSG00000116663 | FBXO6 | F-box protein 6 | 1.4 | 4.5 |
| ENSG00000150337 | FCGR1A | Fc fragment of IgG receptor Ia | 1.3 | 4.5 |
| ENSG00000126262 | FFAR2 | free fatty acid receptor 2 | 1.2 | 7.3 |
| ENSG00000134183 | GNAT2 | G protein subunit alpha transducin 2 | -1.0 | 5.7 |
| ENSG00000189060 | H1F0 | H1.0 linker histone | 1.1 | 5.1 |
| ENSG00000130589 | HELZ2 | helicase with zinc finger 2 | 1.3 | 8.3 |
| ENSG00000138642 | HERC6 | HECT and RLD domain containing E3 ubiquitin protein ligase family member 6 | 1.2 | 5.1 |
| ENSG00000010704 | HFE | homeostatic iron regulator | 1.3 | 4.4 |
| ENSG00000216331 | HIST1H1PS1 | H1.12 linker histone, cluster member pseudogene | 1.5 | 4.4 |
| ENSG00000180573 | HIST1H2AC | H2A clustered histone 6 | 1.1 | 6.5 |
| ENSG00000180596 | HIST1H2BC | H2B clustered histone 4 | 1.0 | 3.0 |
| ENSG00000158373 | HIST1H2BD | H2B clustered histone 5 | 1.1 | 4.5 |
| ENSG00000124635 | HIST1H2BJ | H2B clustered histone 11 | 1.2 | 2.5 |
| ENSG00000278828 | HIST1H3H | H3 clustered histone 10 | 1.1 | 3.8 |
| ENSG00000184678 | HIST2H2BE | H2B clustered histone 21 | 1.0 | 4.6 |
| ENSG00000068079 | IFI35 | interferon induced protein 35 | 1.9 | 6.1 |
| ENSG00000126709 | IFI6 | interferon alpha inducible protein 6 | 2.7 | 8.1 |
| ENSG00000185745 | IFIT1 | interferon induced protein with tetratricopeptide repeats 1 | 2.7 | 5.8 |
| ENSG00000119922 | IFIT2 | interferon induced protein with tetratricopeptide repeats 2 | 1.9 | 6.2 |
| ENSG00000119917 | IFIT3 | interferon induced protein with tetratricopeptide repeats 3 | 2.7 | 6.7 |
| ENSG00000152778 | IFIT5 | interferon induced protein with tetratricopeptide repeats 5 | 1.8 | 4.7 |
| ENSG00000142089 | IFITM3 | interferon induced transmembrane protein 3 | 2.1 | 9.1 |
| ENSG00000211895 | IGHA1 | immunoglobulin heavy constant alpha 1 | 1.7 | 8.8 |
| ENSG00000211890 | IGHA2 | immunoglobulin heavy constant alpha 2 (A2m marker) | 1.1 | 6.5 |
| ENSG00000211896 | IGHG1 | immunoglobulin heavy constant gamma 1 (G1m marker) | 2.0 | 8.6 |
| ENSG00000211893 | IGHG2 | immunoglobulin heavy constant gamma 2 (G2m marker) | 1.3 | 7.0 |
| ENSG00000211897 | IGHG3 | immunoglobulin heavy constant gamma 3 (G3m marker) | 1.6 | 6.0 |
| ENSG00000239951 | IGKV3-20 | immunoglobulin kappa variable 3-20 | 1.3 | 5.1 |
| ENSG00000211677 | IGLC2 | immunoglobulin lambda constant 2 | 1.7 | 7.4 |
| ENSG00000211679 | IGLC3 | immunoglobulin lambda constant 3 (Kern-Oz+ marker) | 1.2 | 5.9 |
| ENSG00000211666 | IGLV2-14 | immunoglobulin lambda variable 2-14 | 1.2 | 3.8 |
| ENSG00000136689 | IL1RN | interleukin 1 receptor antagonist | 2.0 | 5.6 |
| ENSG00000185507 | IRF7 | interferon regulatory factor 7 | 2.2 | 8.2 |
| ENSG00000187608 | ISG15 | ISG15 ubiquitin like modifier | 3.8 | 9.3 |
| ENSG00000173801 | JUP | junction plakoglobin | 2.1 | 5.7 |
| ENSG00000144445 | KANSL1L | KAT8 regulatory NSL complex subunit 1 like | -1.0 | 4.8 |
| ENSG00000108773 | KAT2A | lysine acetyltransferase 2A | 1.1 | 4.2 |
| ENSG00000185909 | KLHDC8B | kelch domain containing 8B | 1.4 | 3.4 |
| ENSG00000089692 | LAG3 | lymphocyte activating 3 | 1.5 | 3.1 |
| ENSG00000002549 | LAP3 | leucine aminopeptidase 3 | 1.6 | 4.8 |
| ENSG00000148346 | LCN2 | lipocalin 2 | 2.1 | 4.2 |
| ENSG00000108679 | LGALS3BP | galectin 3 binding protein | 2.3 | 5.7 |
| ENSG00000168961 | LGALS9 | galectin 9 | 1.2 | 6.5 |
| ENSG00000187116 | LILRA5 | leukocyte immunoglobulin like receptor A5 | 1.1 | 4.0 |
| ENSG00000186818 | LILRB4 | leukocyte immunoglobulin like receptor B4 | 1.1 | 4.6 |
| ENSG00000107798 | LIPA | lipase A, lysosomal acid type | 2.2 | 12.4 |
| ENSG00000238083 | LRRC37A2 | leucine rich repeat containing 37 member A2 | -1.1 | 5.0 |
| ENSG00000160932 | LY6E | lymphocyte antigen 6 family member E | 3.1 | 9.0 |
| ENSG00000204103 | MAFB | MAF bZIP transcription factor B | 1.1 | 5.8 |
| ENSG00000183019 | MCEMP1 | mast cell expressed membrane protein 1 | 1.0 | 2.8 |
| ENSG00000104738 | MCM4 | minichromosome maintenance complex component 4 | 1.0 | 2.3 |
| ENSG00000100985 | MMP9 | matrix metallopeptidase 9 | 1.2 | 6.8 |
| ENSG00000155363 | MOV10 | Mov10 RISC complex RNA helicase | 1.4 | 5.5 |
| ENSG00000125148 | MT2A | metallothionein 2A | 2.9 | 6.2 |
| ENSG00000157601 | MX1 | MX dynamin like GTPase 1 | 2.4 | 8.3 |
| ENSG00000183486 | MX2 | MX dynamin like GTPase 2 | 1.3 | 7.3 |
| ENSG00000170476 | MZB1 | marginal zone B and B1 cell specific protein | 1.6 | 4.1 |
| ENSG00000130202 | NECTIN2 | nectin cell adhesion molecule 2 | 1.5 | 3.6 |
| ENSG00000165030 | NFIL3 | nuclear factor, interleukin 3 regulated | 1.1 | 3.8 |
| ENSG00000196358 | NTNG2 | netrin G2 | 1.3 | 6.7 |
| ENSG00000089127 | OAS1 | 2'-5'-oligoadenylate synthetase 1 | 3.0 | 7.0 |
| ENSG00000111335 | OAS2 | 2'-5'-oligoadenylate synthetase 2 | 2.6 | 6.5 |
| ENSG00000111331 | OAS3 | 2'-5'-oligoadenylate synthetase 3 | 2.9 | 7.9 |
| ENSG00000135114 | OASL | 2'-5'-oligoadenylate synthetase like | 3.1 | 6.5 |
| ENSG00000177989 | ODF3B | outer dense fiber of sperm tails 3B | 1.2 | 6.7 |
| ENSG00000059378 | PARP12 | poly(ADP-ribose) polymerase family member 12 | 1.4 | 5.8 |
| ENSG00000138496 | PARP9 | poly(ADP-ribose) polymerase family member 9 | 1.1 | 5.6 |
| ENSG00000145287 | PLAC8 | placenta associated 8 | 1.1 | 5.1 |
| ENSG00000188313 | PLSCR1 | phospholipid scramblase 1 | 1.9 | 4.3 |
| ENSG00000140464 | PML | promyelocytic leukemia | 1.3 | 6.7 |
| ENSG00000197549 | PRAMENP | PRAME N-terminal like, pseudogene | 1.1 | 6.0 |
| ENSG00000100567 | PSMA3 | proteasome 20S subunit alpha 3 | -1.1 | 4.9 |
| ENSG00000119707 | RBM25 | RNA binding motif protein 25 | -1.1 | 4.0 |
| ENSG00000169385 | RNASE2 | ribonuclease A family member 2 | 1.4 | 3.0 |
| ENSG00000234719 | RP11-166B2.1 | nuclear pore complex interacting protein family member B2 | 1.1 | 4.8 |
| ENSG00000279296 | RP11-609D21.3 | p53 regulation associated lncRNA | 1.2 | 8.0 |
| ENSG00000250349 | RP5-972B16.2 | novel proline rich Gla (G-carboxyglutamic acid) 1 (PRRG1) and tetraspanin 7 (TSPAN7) protein | -1.2 | 5.7 |
| ENSG00000188282 | RUFY4 | RUN and FYVE domain containing 4 | 1.6 | 7.9 |
| ENSG00000163220 | S100A9 | S100 calcium binding protein A9 | 1.0 | 12.1 |
| ENSG00000130066 | SAT1 | spermidine/spermine N1-acetyltransferase 1 | 1.1 | 7.1 |
| ENSG00000130489 | SCO2 | cytochrome c oxidase assembly protein | 1.3 | 6.5 |
| ENSG00000149131 | SERPING1 | serpin family G member 1 | 3.1 | 5.6 |
| ENSG00000149212 | SESN3 | sestrin 3 | -1.0 | 3.7 |
| ENSG00000164054 | SHISA5 | shisa family member 5 | 1.3 | 8.1 |
| ENSG00000088827 | SIGLEC1 | sialic acid binding Ig like lectin 1 | 3.8 | 7.1 |
| ENSG00000004939 | SLC4A1 | solute carrier family 4 member 1 (Diego blood group) | -1.1 | 5.8 |
| ENSG00000183023 | SLC8A1 | solute carrier family 8 member A1 | -1.0 | 4.7 |
| ENSG00000214872 | SMTNL1 | smoothelin like 1 | 2.4 | 7.7 |
| ENSG00000185482 | STAC3 | SH3 and cysteine rich domain 3 | 1.1 | 2.8 |
| ENSG00000134809 | TIMM10 | translocase of inner mitochondrial membrane 10 | 1.1 | 3.0 |
| ENSG00000121858 | TNFSF10 | TNF superfamily member 10 | 1.3 | 5.0 |
| ENSG00000135148 | TRAFD1 | TRAF-type zinc finger domain containing 1 | 1.1 | 5.8 |
| ENSG00000132274 | TRIM22 | tripartite motif containing 22 | 1.3 | 6.3 |
| ENSG00000132256 | TRIM5 | tripartite motif containing 5 | 1.1 | 10.9 |
| ENSG00000185880 | TRIM69 | tripartite motif containing 69 | 1.1 | 4.2 |
| ENSG00000123297 | TSFM | Ts translation elongation factor, mitochondrial | -1.1 | 5.2 |
| ENSG00000140830 | TXNL4B | thioredoxin like 4B | 1.1 | 6.8 |
| ENSG00000025708 | TYMP | thymidine phosphorylase | 1.1 | 8.4 |
| ENSG00000156587 | UBE2L6 | ubiquitin conjugating enzyme E2 L6 | 1.5 | 6.6 |
| ENSG00000168899 | VAMP5 | vesicle associated membrane protein 5 | 1.1 | 4.7 |
| ENSG00000132530 | XAF1 | XIAP associated factor 1 | 1.5 | 4.6 |
| ENSG00000124256 | ZBP1 | Z-DNA binding protein 1 | 2.0 | 6.3 |
| ENSG00000141664 | ZCCHC2 | zinc finger CCHC-type containing 2 | 1.0 | 4.8 |
| ENSG00000162714 | ZNF496 | zinc finger protein 496 | 1.0 | 4.0 |
| ENSG00000196357 | ZNF565 | zinc finger protein 565 | -1.0 | 5.6 |

Supplementary Table 4. List of genes that were induced in both our whole blood signatures and reported signatures of respiratory syncytial virus (RSV) [1]

| Ensembl ID | Gene name | Gene description |
| --- | --- | --- |
| ENSG00000187608 | ISG15 | ISG15 ubiquitin like modifier |
| ENSG00000126262 | FFAR2 | free fatty acid receptor 2 |
| ENSG00000187116 | LILRA5 | leukocyte immunoglobulin like receptor A5 |
| ENSG00000135114 | OASL | 2'-5'-oligoadenylate synthetase like |
| ENSG00000150337 | FCGR1A | Fc fragment of IgG receptor Ia |
| ENSG00000111331 | OAS3 | 2'-5'-oligoadenylate synthetase 3 |
| ENSG00000185745 | IFIT1 | interferon induced protein with tetratricopeptide repeats 1 |
| ENSG00000185507 | IRF7 | interferon regulatory factor 7 |
| ENSG00000148926 | ADM | adrenomedullin |
| ENSG00000119922 | IFIT2 | interferon induced protein with tetratricopeptide repeats 2 |
| ENSG00000100985 | MMP9 | matrix metallopeptidase 9 |
| ENSG00000188313 | PLSCR1 | phospholipid scramblase 1 |
| ENSG00000119917 | IFIT3 | interferon induced protein with tetratricopeptide repeats 3 |
| ENSG00000136689 | IL1RN | interleukin 1 receptor antagonist |

Supplementary Table 5. List of genes that were induced in both our whole blood signatures and reported signatures of Influenza patients with severe or moderate symptoms.[2]

| Ensembl ID | Gene name | Gene description |
| --- | --- | --- |
| ENSG00000125148 | MT2A | metallothionein 2A |
| ENSG00000183019 | MCEMP1 | mast cell expressed membrane protein 1 |
| ENSG00000163220 | S100A9 | S100 calcium binding protein A9 |
| ENSG00000124256 | ZBP1 | Z-DNA binding protein 1 |
| ENSG00000121858 | TNFSF10 | TNF superfamily member 10 |
| ENSG00000089127 | OAS1 | 2'-5'-oligoadenylate synthetase 1 |
| ENSG00000140379 | BCL2A1 | BCL2 related protein A1 |
| ENSG00000196358 | NTNG2 | netrin G2 |
| ENSG00000132530 | XAF1 | XIAP associated factor 1 |
| ENSG00000187608 | ISG15 | ISG15 ubiquitin like modifier |
| ENSG00000068079 | IFI35 | interferon induced protein 35 |
| ENSG00000160932 | LY6E | lymphocyte antigen 6 family member E |
| ENSG00000088827 | SIGLEC1 | sialic acid binding Ig like lectin 1 |
| ENSG00000187116 | LILRA5 | leukocyte immunoglobulin like receptor A5 |
| ENSG00000148346 | LCN2 | lipocalin 2 |
| ENSG00000135114 | OASL | 2'-5'-oligoadenylate synthetase like |
| ENSG00000170476 | MZB1 | marginal zone B and B1 cell specific protein |
| ENSG00000073737 | DHRS9 | dehydrogenase/reductase 9 |
| ENSG00000150337 | FCGR1A | Fc fragment of IgG receptor Ia |
| ENSG00000169385 | RNASE2 | ribonuclease A family member 2 |
| ENSG00000134809 | TIMM10 | translocase of inner mitochondrial membrane 10 |
| ENSG00000111331 | OAS3 | 2'-5'-oligoadenylate synthetase 3 |
| ENSG00000185745 | IFIT1 | interferon induced protein with tetratricopeptide repeats 1 |
| ENSG00000185507 | IRF7 | interferon regulatory factor 7 |
| ENSG00000180573 | HIST1H2AC | H2A clustered histone 6 |
| ENSG00000133106 | EPSTI1 | epithelial stromal interaction 1 |
| ENSG00000149131 | SERPING1 | serpin family G member 1 |
| ENSG00000148926 | ADM | adrenomedullin |
| ENSG00000116663 | FBXO6 | F-box protein 6 |
| ENSG00000119922 | IFIT2 | interferon induced protein with tetratricopeptide repeats 2 |
| ENSG00000055332 | EIF2AK2 | eukaryotic translation initiation factor 2 alpha kinase 2 |
| ENSG00000141664 | ZCCHC2 | zinc finger CCHC-type containing 2 |
| ENSG00000100985 | MMP9 | matrix metallopeptidase 9 |
| ENSG00000111335 | OAS2 | 2'-5'-oligoadenylate synthetase 2 |
| ENSG00000188313 | PLSCR1 | phospholipid scramblase 1 |
| ENSG00000157601 | MX1 | MX dynamin like GTPase 1 |
| ENSG00000130489 | SCO2 | cytochrome c oxidase assembly protein |
| ENSG00000119917 | IFIT3 | interferon induced protein with tetratricopeptide repeats 3 |
| ENSG00000136689 | IL1RN | interleukin 1 receptor antagonist |
| ENSG00000126709 | IFI6 | interferon alpha inducible protein 6 |

Supplementary Table 6. List of 141 GO pathways that were overrepresented in our COVID-19 blood signature; also overrepresented in RSV ($) and Influenza (#).

| GO Term | GO pathways |
| --- | --- |
| GO:0002252 | immune effector process $# |
| GO:0002221 | pattern recognition receptor signaling pathway $# |
| GO:0051707 | response to other organism $# |
| GO:0034097 | response to cytokine $# |
| GO:0032479 | regulation of type I interferon production $# |
| GO:0071310 | cellular response to organic substance $# |
| GO:0042221 | response to chemical $# |
| GO:0007165 | signal transduction $# |
| GO:0098542 | defense response to other organism $# |
| GO:0009605 | response to external stimulus $# |
| GO:0006952 | defense response $# |
| GO:0045071 | negative regulation of viral genome replication $# |
| GO:0009615 | response to virus $# |
| GO:0031348 | negative regulation of defense response $# |
| GO:0019221 | cytokine-mediated signaling pathway $# |
| GO:0045088 | regulation of innate immune response $# |
| GO:0032020 | ISG15-protein conjugation $# |
| GO:0050776 | regulation of immune response $# |
| GO:0048519 | negative regulation of biological process $# |
| GO:0052548 | regulation of endopeptidase activity $# |
| GO:0043900 | regulation of multi-organism process $# |
| GO:0042742 | defense response to bacterium $# |
| GO:0001817 | regulation of cytokine production $# |
| GO:0045824 | negative regulation of innate immune response $# |
| GO:0002699 | positive regulation of immune effector process $# |
| GO:0045637 | regulation of myeloid cell differentiation $# |
| GO:0045087 | innate immune response $# |
| GO:0032940 | secretion by cell $# |
| GO:1903706 | regulation of hemopoiesis $# |
| GO:0051607 | defense response to virus $# |
| GO:0050789 | regulation of biological process $# |
| GO:0048583 | regulation of response to stimulus $# |
| GO:0043312 | neutrophil degranulation $# |
| GO:0006953 | acute-phase response $# |
| GO:0065007 | biological regulation $# |
| GO:0060337 | type I interferon signaling pathway $# |
| GO:0051239 | regulation of multicellular organismal process $# |
| GO:0050896 | response to stimulus $# |
| GO:0002831 | regulation of response to biotic stimulus $# |
| GO:0071345 | cellular response to cytokine stimulus $# |
| GO:0060333 | interferon-gamma-mediated signaling pathway $# |
| GO:0048584 | positive regulation of response to stimulus $# |
| GO:0035457 | cellular response to interferon-alpha $# |
| GO:0009617 | response to bacterium $# |
| GO:0006955 | immune response $# |
| GO:0007154 | cell communication $# |
| GO:0002682 | regulation of immune system process $# |
| GO:0051240 | positive regulation of multicellular organismal process $# |
| GO:0010033 | response to organic substance $# |
| GO:0050794 | regulation of cellular process $# |
| GO:0050688 | regulation of defense response to virus $# |
| GO:0050778 | positive regulation of immune response $# |
| GO:0050691 | regulation of defense response to virus by host $# |
| GO:0002376 | immune system process $# |
| GO:0001818 | negative regulation of cytokine production $# |
| GO:0051716 | cellular response to stimulus $# |
| GO:0006954 | inflammatory response $# |
| GO:0031347 | regulation of defense response $# |
| GO:0034341 | response to interferon-gamma $# |
| GO:0002443 | leukocyte mediated immunity $# |
| GO:0007166 | cell surface receptor signaling pathway $# |
| GO:0006950 | response to stress $# |
| GO:0080134 | regulation of response to stress $# |
| GO:0045055 | regulated exocytosis $# |
| GO:0002697 | regulation of immune effector process $# |
| GO:0002684 | positive regulation of immune system process $# |
| GO:0051704 | multi-organism process $# |
| GO:0045089 | positive regulation of innate immune response $# |
| GO:0002694 | regulation of leukocyte activation $# |
| GO:0035456 | response to interferon-beta $# |
| GO:1902105 | regulation of leukocyte differentiation $ |
| GO:0023052 | signaling $ |
| GO:0002703 | regulation of leukocyte mediated immunity $ |
| GO:0006887 | exocytosis $ |
| GO:0071346 | cellular response to interferon-gamma $ |
| GO:0002698 | negative regulation of immune effector process $ |
| GO:0045589 | regulation of regulatory T cell differentiation $ |
| GO:0061844 | antimicrobial humoral immune response mediated by antimicrobial peptide # |
| GO:0006878 | cellular copper ion homeostasis # |
| GO:0006959 | humoral immune response # |
| GO:0051092 | positive regulation of NF-kappaB transcription factor activity # |
| GO:0051091 | positive regulation of DNA-binding transcription factor activity # |
| GO:0046914 | transition metal ion binding # |
| GO:0042448 | progesterone metabolic process # |
| GO:0043901 | negative regulation of multi-organism process # |
| GO:2001235 | positive regulation of apoptotic signaling pathway # |
| GO:0019730 | antimicrobial humoral response # |
| GO:0009987 | cellular process # |
| GO:0050801 | ion homeostasis # |
| GO:0002683 | negative regulation of immune system process # |
| GO:0070887 | cellular response to chemical stimulus # |
| GO:0002227 | innate immune response in mucosa # |
| GO:0035455 | response to interferon-alpha # |
| GO:0003725 | double-stranded RNA binding # |
| GO:0002523 | leukocyte migration involved in inflammatory response # |
| GO:0001730 | 2'-5'-oligoadenylate synthetase activity # |
| GO:0050830 | defense response to Gram-positive bacterium # |
| GO:0006875 | cellular metal ion homeostasis # |
| GO:0046982 | protein heterodimerization activity # |
| GO:0042592 | homeostatic process # |
| GO:0006915 | apoptotic process # |
| GO:0044419 | interspecies interaction between organisms # |
| GO:0032480 | negative regulation of type I interferon production # |
| GO:0005488 | binding # |
| GO:0043281 | regulation of cysteine-type endopeptidase activity involved in apoptotic process # |
| GO:0019731 | antibacterial humoral response # |
| GO:0048878 | chemical homeostasis # |
| GO:0060700 | regulation of ribonuclease activity # |
| GO:0046916 | cellular transition metal ion homeostasis # |
| GO:0042802 | identical protein binding # |
| GO:0002385 | mucosal immune response # |
| GO:0050792 | regulation of viral process |
| GO:0033003 | regulation of mast cell activation |
| GO:0016032 | viral process |
| GO:0007005 | mitochondrion organization |
| GO:0006334 | nucleosome assembly |
| GO:0050777 | negative regulation of immune response |
| GO:0061025 | membrane fusion |
| GO:1903900 | regulation of viral life cycle |
| GO:1903901 | negative regulation of viral life cycle |
| GO:0042269 | regulation of natural killer cell mediated cytotoxicity |
| GO:2000026 | regulation of multicellular organismal development |
| GO:2001267 | regulation of cysteine-type endopeptidase activity involved in apoptotic signaling pathway |
| GO:0003374 | dynamin family protein polymerization involved in mitochondrial fission |
| GO:0033212 | iron import into cell |
| GO:0008219 | cell death |
| GO:0046596 | regulation of viral entry into host cell |
| GO:0032880 | regulation of protein localization |
| GO:0051241 | negative regulation of multicellular organismal process |
| GO:0002704 | negative regulation of leukocyte mediated immunity |
| GO:0019060 | intracellular transport of viral protein in host cell |
| GO:0042771 | intrinsic apoptotic signaling pathway in response to DNA damage by p53 class mediator |
| GO:0045591 | positive regulation of regulatory T cell differentiation |
| GO:0008630 | intrinsic apoptotic signaling pathway in response to DNA damage |
| GO:0016567 | protein ubiquitination |
| GO:0012501 | programmed cell death |
| GO:0065003 | protein-containing complex assembly |
| GO:1900180 | regulation of protein localization to nucleus |
| GO:0048585 | negative regulation of response to stimulus |
| GO:0071103 | DNA conformation change |
| GO:1900122 | positive regulation of receptor binding |

Supplementary Table 7. List of 11 KEGG pathways that were overrepresented in our COVID-19 blood signature; also overrepresented in RSV ($) and Influenza (#).

| KEGG Term | KEGG pathway |
| --- | --- |
| hsa05164 | Influenza A $# |
| hsa05322 | Systemic lupus erythematosus $# |
| hsa05202 | Transcriptional misregulation in cancer $# |
| hsa05160 | Hepatitis C $# |
| hsa05162 | Measles # |
| hsa04217 | Necroptosis # |
| hsa05168 | Herpes simplex infection # |
| hsa05167 | Kaposi's sarcoma-associated herpesvirus infection |
| hsa05203 | Viral carcinogenesis |
| hsa05221 | Acute myeloid leukemia |
| hsa05219 | Bladder cancer |

Supplementary Table 8. List of 78 overrepresented GO pathways found in COVID-19 Chinese patients but not in Indian patients (relative to controls).

| GO Term | GO pathways |
| --- | --- |
| GO:0002702 | positive regulation of production of molecular mediator of immune response |
| GO:0005488 | Binding |
| GO:0051099 | positive regulation of binding |
| GO:0002252 | immune effector process |
| GO:0009987 | cellular process |
| GO:0002757 | immune response-activating signal transduction |
| GO:0051707 | response to other organism |
| GO:0061844 | antimicrobial humoral immune response mediated by antimicrobial peptide |
| GO:0002764 | immune response-regulating signaling pathway |
| GO:0019864 | IgG binding |
| GO:0019731 | antibacterial humoral response |
| GO:0050729 | positive regulation of inflammatory response |
| GO:0045321 | leukocyte activation |
| GO:0016477 | cell migration |
| GO:0031347 | regulation of defense response |
| GO:0016192 | vesicle-mediated transport |
| GO:0006959 | humoral immune response |
| GO:0042742 | defense response to bacterium |
| GO:0042590 | antigen processing and presentation of exogenous peptide antigen via MHC class I |
| GO:0002227 | innate immune response in mucosa |
| GO:0070887 | cellular response to chemical stimulus |
| GO:0034097 | response to cytokine |
| GO:0035662 | Toll-like receptor 4 binding |
| GO:0065003 | protein-containing complex assembly |
| GO:0071310 | cellular response to organic substance |
| GO:0071345 | cellular response to cytokine stimulus |
| GO:0002443 | leukocyte mediated immunity |
| GO:0060326 | cell chemotaxis |
| GO:0046916 | cellular transition metal ion homeostasis |
| GO:0006898 | receptor-mediated endocytosis |
| GO:0019763 | immunoglobulin receptor activity |
| GO:0032119 | sequestering of zinc ion |
| GO:0042221 | response to chemical |
| GO:0006950 | response to stress |
| GO:0043933 | protein-containing complex subunit organization |
| GO:0032940 | secretion by cell |
| GO:0050786 | RAGE receptor binding |
| GO:0080134 | regulation of response to stress |
| GO:0032602 | chemokine production |
| GO:0050900 | leukocyte migration |
| GO:0098542 | defense response to other organism |
| GO:0006911 | phagocytosis, engulfment |
| GO:0050727 | regulation of inflammatory response |
| GO:0009605 | response to external stimulus |
| GO:0007159 | leukocyte cell-cell adhesion |
| GO:0050544 | arachidonic acid binding |
| GO:0010033 | response to organic substance |
| GO:0002700 | regulation of production of molecular mediator of immune response |
| GO:0002523 | leukocyte migration involved in inflammatory response |
| GO:0006887 | Exocytosis |
| GO:0002718 | regulation of cytokine production involved in immune response |
| GO:0030595 | leukocyte chemotaxis |
| GO:0032392 | DNA geometric change |
| GO:0002274 | myeloid leukocyte activation |
| GO:0038093 | Fc receptor signaling pathway |
| GO:0018119 | peptidyl-cysteine S-nitrosylation |
| GO:0006268 | DNA unwinding involved in DNA replication |
| GO:0006334 | nucleosome assembly |
| GO:0070488 | neutrophil aggregation |
| GO:0050778 | positive regulation of immune response |
| GO:0051704 | multi-organism process |
| GO:0043388 | positive regulation of DNA binding |
| GO:0098657 | import into cell |
| GO:0048583 | regulation of response to stimulus |
| GO:0050830 | defense response to Gram-positive bacterium |
| GO:0043312 | neutrophil degranulation |
| GO:0071103 | DNA conformation change |
| GO:0051101 | regulation of DNA binding |
| GO:0019221 | cytokine-mediated signaling pathway |
| GO:2001235 | positive regulation of apoptotic signaling pathway |
| GO:0045088 | regulation of innate immune response |
| GO:0033212 | iron import into cell |
| GO:0030593 | neutrophil chemotaxis |
| GO:1900122 | positive regulation of receptor binding |
| GO:0002720 | positive regulation of cytokine production involved in immune response |
| GO:0002385 | mucosal immune response |
| GO:0045089 | positive regulation of innate immune response |
| GO:0019730 | antimicrobial humoral response |

Supplementary Table 9. List of 21 overrepresented GO pathways found in COVID-19 Indian patients but not in Chinese patients (relative to controls).

| GO Term | GO pathway |
| --- | --- |
| GO:0045595 | regulation of cell differentiation |
| GO:2000484 | positive regulation of interleukin-8 secretion |
| GO:0045954 | positive regulation of natural killer cell mediated cytotoxicity |
| GO:0002690 | positive regulation of leukocyte chemotaxis |
| GO:0051239 | regulation of multicellular organismal process |
| GO:0071622 | regulation of granulocyte chemotaxis |
| GO:0050856 | regulation of T cell receptor signaling pathway |
| GO:0032703 | negative regulation of interleukin-2 production |
| GO:0006935 | Chemotaxis |
| GO:0010759 | positive regulation of macrophage chemotaxis |
| GO:0032880 | regulation of protein localization |
| GO:0050860 | negative regulation of T cell receptor signaling pathway |
| GO:0051241 | negative regulation of multicellular organismal process |
| GO:0045596 | negative regulation of cell differentiation |
| GO:0042269 | regulation of natural killer cell mediated cytotoxicity |
| GO:0019835 | Cytolysis |
| GO:2000026 | regulation of multicellular organismal development |
| GO:0045591 | positive regulation of regulatory T cell differentiation |
| GO:0046135 | pyrimidine nucleoside catabolic process |
| GO:0015671 | oxygen transport |
| GO:0050793 | regulation of developmental process |

Supplementary Table 10. List of 10 GO and 2 KEGG pathways that were enriched in the final disease signature (overlap of discovery and validation) and COVID-19 (single-group analysis; not found in RSV and Influenza).

| GO or KEGG Term | Pathway |
| --- | --- |
| GO:0012501 | programmed cell death |
| GO:0050777 | negative regulation of immune response |
| GO:1903900 | regulation of viral life cycle |
| GO:1903901 | negative regulation of viral life cycle |
| GO:0051241 | negative regulation of multicellular organismal process |
| GO:0007005 | mitochondrion organization |
| GO:0042771 | intrinsic apoptotic signaling pathway in response to DNA damage by p53 class mediator |
| GO:0045591 | positive regulation of regulatory T cell differentiation |
| GO:0003374 | dynamin family protein polymerization involved in mitochondrial fission |
| GO:0008630 | intrinsic apoptotic signaling pathway in response to DNA damage |
| hsa05203 | Viral carcinogenesis |
| hsa05221 | Acute myeloid leukemia |

Supplementary Figure 1. PCA analysis of the transcriptome data from all COVID-19 cases and controls. RED: cases; BLACK: controls


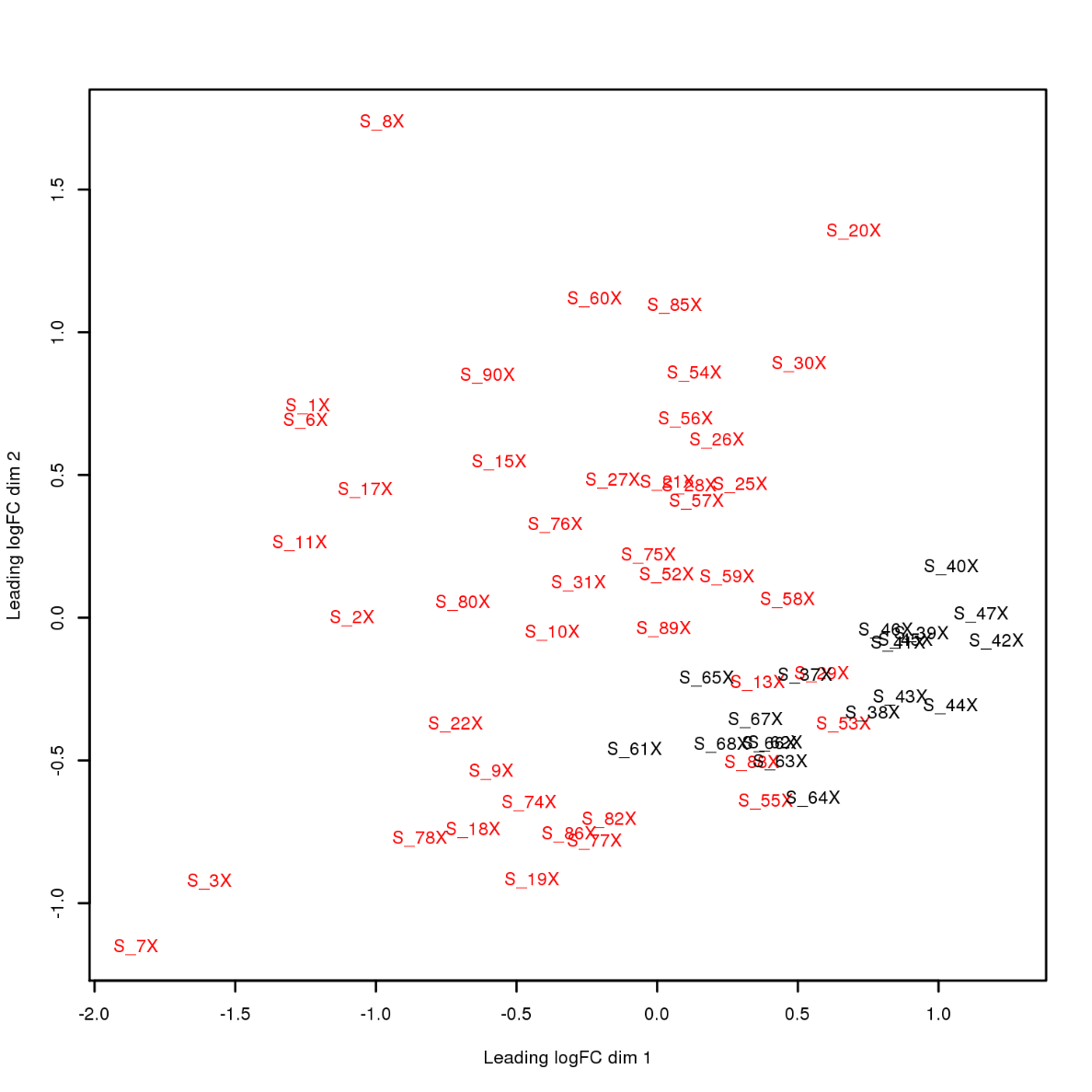


Supplementary Figure 2. Key bioinformatics parameters for sequencing read mapping, transcript counting and differential analysis by STAR, HTseq and EdgeR respectively.


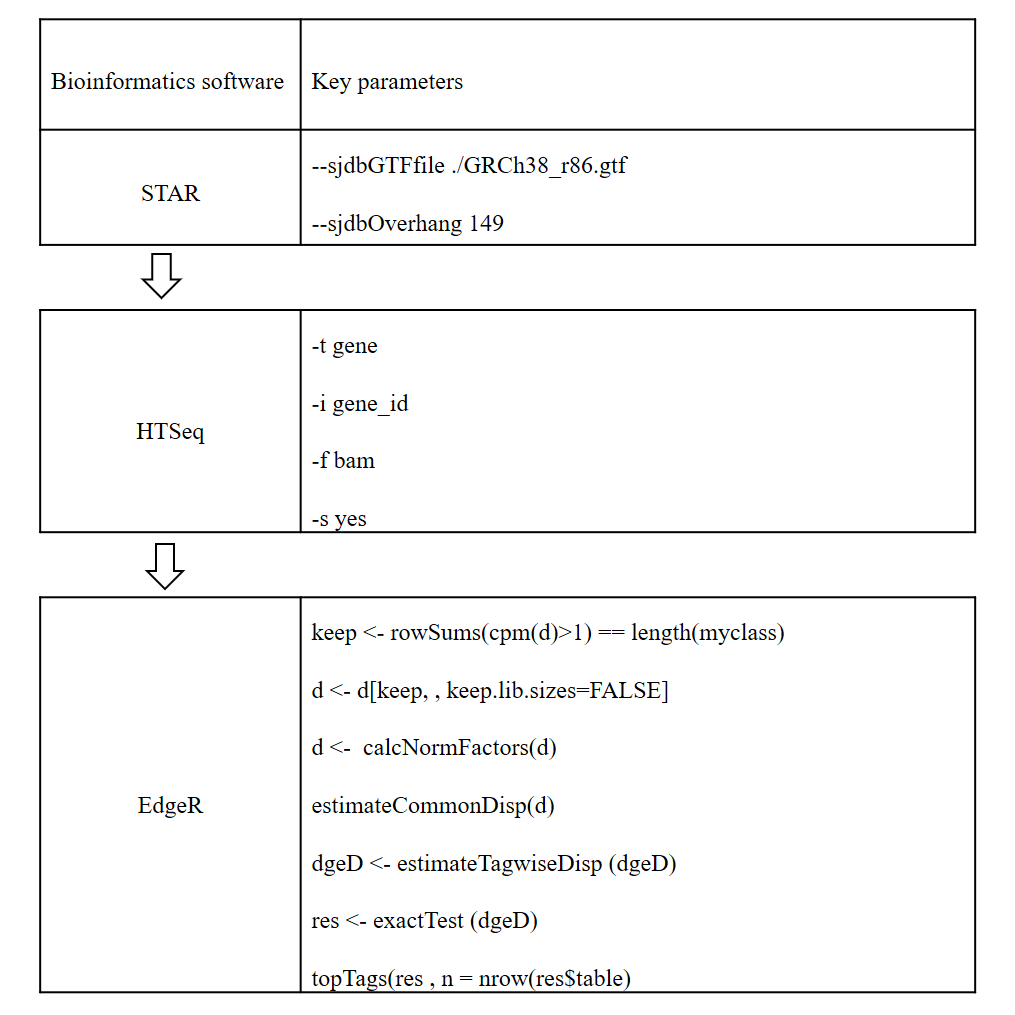


Supplementary Figure 3. Relative gene expression values of the 14 overlapping induced genes from published RSV and COVID-19 dataset in this study.

Supplementary Figure 4. Relative gene expression values of the 40 overlapping induced genes from published Influenza and COVID-19 dataset in this study.

References

1. Do LAH, Pellet J, van Doorn HR, Tran AT, Nguyen BH, Tran TTL, Tran QH, Vo QB, Tran Dac NA, Trinh HN: **Host transcription profile in nasal epithelium and whole blood of hospitalized children under 2 years of age with respiratory syncytial virus infection**. *The Journal of infectious diseases* 2018, **217**(1):134-146.

2. Zerbib Y, Jenkins EK, Shojaei M, Meyers AF, Ho J, Ball TB, Keynan Y, Pisipati A, Kumar A, Kumar A: **Pathway mapping of leukocyte transcriptome in influenza patients reveals distinct pathogenic mechanisms associated with progression to severe infection**. *BMC medical genomics* 2020, **13**(1):1-13.
